# Structural Bases for Hesperetin Derivatives: Inhibition of Protein Tyrosine Phosphatase 1B, Kinetics Mechanism and Molecular Docking Study

**DOI:** 10.3390/molecules26247433

**Published:** 2021-12-08

**Authors:** Md Yousof Ali, Susoma Jannat, Hyun-Ah Jung, Jae-Sue Choi

**Affiliations:** 1 Department of Physiology and Pharmacology, Hotchkiss Brain Institute and Alberta Children’s Hospital Research Institute, Cumming School of Medicine, University of Calgary, Calgary, AB T2N 4N1, Canada; mdyousof.ali@ucalgary.ca; 2 Department of Biochemistry and Molecular Biology, University of Calgary, AB T2N 1N4, Canada; jannatacct@gmail.com; 3 Department of Food Science and Human Nutrition, Jeonbuk National University, Jeonju 54896, Korea; 4 Department of Food and Life Science, Pukyong National University, Busan 48513, Korea

**Keywords:** hesperetin derivatives, PTP1B, hesperetin 5-*O*-glucoside, structure-activity relationship, molecular docking

## Abstract

In the present study, we investigated the structure-activity relationship of naturally occurring hesperetin derivatives, as well as the effects of their glycosylation on the inhibition of diabetes-related enzyme systems, protein tyrosine phosphatase 1B (PTP1B) and α-glycosidase. Among the tested hesperetin derivatives, hesperetin 5-*O*-glucoside, a single-glucose-containing flavanone glycoside, significantly inhibited PTP1B with an IC_50_ value of 37.14 ± 0.07 µM. Hesperetin, which lacks a sugar molecule, was the weakest inhibitor compared to the reference compound, ursolic acid (IC_50_ = 9.65 ± 0.01 µM). The most active flavanone hesperetin 5-*O*-glucoside suggested that the position of a sugar moiety at the C-5-position influences the PTP1B inhibition. It was observed that the ability to inhibit PTP1B is dependent on the nature, position, and number of sugar moieties in the flavonoid structure, as well as conjugation. In the kinetic study of PTP1B enzyme inhibition, hesperetin 5-*O*-glucoside led to mixed-type inhibition. Molecular docking studies revealed that hesperetin 5-*O*-glucoside had a higher binding affinity with key amino residues, suggesting that this molecule best fits the PTP1B allosteric site cavity. The data reported here support hesperetin 5-*O*-glucoside as a hit for the design of more potent and selective inhibitors against PTP1B in the search for a new anti-diabetic treatment.

## 1. Introduction

Diabetes mellitus (DM) has emerged as a major threat to human health, and the expected number of diabetic patients will exceed 642 million by 2045 globally [1,2]. DM is a chronic metabolic disease, which is characterized by consistently high sugar levels in the bloodstream due to insulin deficiency, insulin resistance, or both [2,3]. Excessive glucose in the blood damages blood vessels and nerves, leading to various diseases, such as hypertension, cardiovascular disease, blindness, stroke, amputations, kidney, and dental diseases [2,4,5]. Despite the fact that there are various treatment options, achieving optimal glycemic control without side effects is difficult.

Recent studies on the pathological mechanism revealed that DM has a close relation to the protein tyrosine phosphatase (PTP) family, which plays a pivotal role in the regulation of insulin function by dephosphorylating the tyrosine residues of proteins [6]. Among the PTP family, PTP1B is a critical member and is in charge of insulin and leptin signaling pathways, and is a negative regulator of the insulin receptor (IR) signal transduction pathway that leads to insulin resistance, which makes this enzyme a promising therapeutic target to manage DM [7,8]. In insulin signaling, PTP1B is known to dephosphorylate the activated insulin receptor and insulin receptor substrates to attenuate the cellular response to insulin binding [7,9]. Mice with PTP1B gene knockouts have enhanced insulin sensitivity and low body weight, even when fed a high-fat diet [10]. For these reasons, PTP1B has become an active therapeutic target for the treatment of type II diabetes. Since PTP1B was discovered more than 25 years ago, it has proven to play a critical role in multiple cellular processes, particularly glucose uptake, body mass regulation, motility, and proliferation [7,11]. Considering the rationale above, PTP1B inhibition may modulate DM, and thus, is an outstanding target for the treatment of this epidemic disease. Thus, PTP1B is an attractive target in the development of new treatments for DM and other related metabolic syndromes.

Flavonoids are phenolic compounds widely distributed in the plant kingdom and are important components of the human diet. Different in vitro and in vivo studies have reported the possible role of citrus fruits and their phenolic components in attenuating metabolic syndrome [12]. Flavonoids are reactive and secondary metabolites abundant in plant-derived foods, particularly fruits, seeds, and vegetables [11,13,14]. The natural flavonoids almost all exist as their *O*-glycoside or *C*-glycoside forms in plants. There are several reports that flavonoids can regulate or modulate blood glucose levels after food intake [12,14,15]. Some of these flavonoids can directly induce the secretion of insulin from pancreatic cells in ex vivo assays, but this effect may not translate to in vivo effectiveness because of their low serum bioavailability [12,16]. Flavonoids like hesperidin, neohesperidin, hesperetin, hesperetin 7-*O*-glucoside, and hesperetin 5-*O*-glucoside are naturally occurring bioflavonoids found abundantly in citrus fruits [17]. Hesperidin, a flavanone glycoside, possesses antioxidant, anti-inflammatory, antifungal, antiviral, anti-cancer, anti-Alzheimer, anti-hypotensive, neuroprotective, and vasodilator activities [18,19,20,21,22]. Neohesperidin has anti-diabetic, antioxidant, anti-gastritis, anti-cancer, neuroprotective, anti-inflammatory, and anti-Alzheimer activities [23,24,25,26,27,28]. Moreover, hesperetin and its two glycoside hesperetin 5-*O*-glucoside, and hesperetin 5-*O*-glucoside exhibit antioxidant, antihyperlipidemic, anti-allergic, anti-bacterial, neuroprotective, anti-cancer, anti-inflammatory activities [29,30,31,32,33,34].

The primary goal of this study is to examine the effects of the aglycone hesperetin and its various glycosides on the inhibition of diabetes-related enzyme system (PTP1B) and to create an accurate structure-activity link. Molecular docking simulations were also applied to complement the inhibitory activity studies and to predict the binding model of the selected hesperetin derivatives to the three-dimensional structure of PTP1B. We also performed enzyme kinetic analyses of the flavonoids using Lineweaver−Burk plots to confirm the type of enzymatic inhibition.

## 2. Results

### 2.1. Inhibition of PTP1B by Hesperetin Derivatives

**Figure 1:**
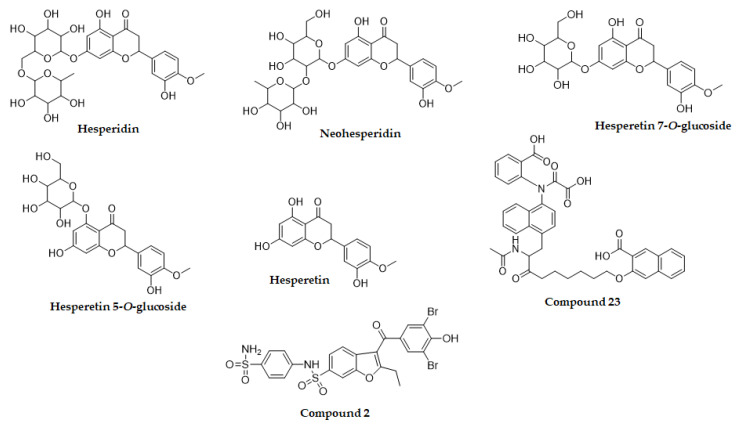
Chemical structures of hesperetin and its glycosylated derivatives.

To evaluate the anti-diabetic activity of hesperetin derivatives, the inhibitory potential of these flavonoids against PTP1B was investigated (Figure 1 and Table 1). Among the tested flavonoids, hesperetin 5-*O*-glucoside and hesperidin exhibited significant PTP1B inhibitory activity with IC_50_ values of 37.14 ± 0.07 and 58.15 ± 4.18 μM, respectively, compared to the IC_50_ value of ursolic acid (9.65 ± 0.01 μM). In addition, neohesperidin and hesperetin displayed weak PTP1B inhibitory activity with corresponding IC_50_ values of 143.63 ± 3.04 and 288.01 ± 7.98 μM, respectively, while hesperetin 7-*O*-glucoside was inactive in the PTP1B inhibitory assay.

### 2.2. Enzyme Kinetics of PTP1B Inhibition

**Figure 2:**
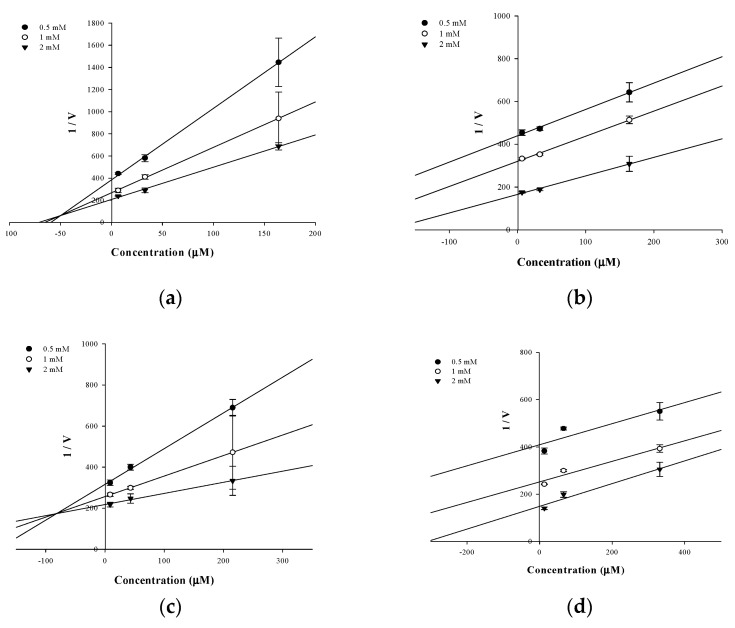
Dixon-plots of the inhibition of PTP1B by the compounds. The results showed the effects of the presence of different concentrations of the substrate (0.5 mM (●), 1 mM (○) and 2 mM (▼) for hesperidin (**a**), neohesperidin (**b**), hesperetin 5-*O*-glucoside (**c**) and hesperetin (**d**).

**Figure 3:**
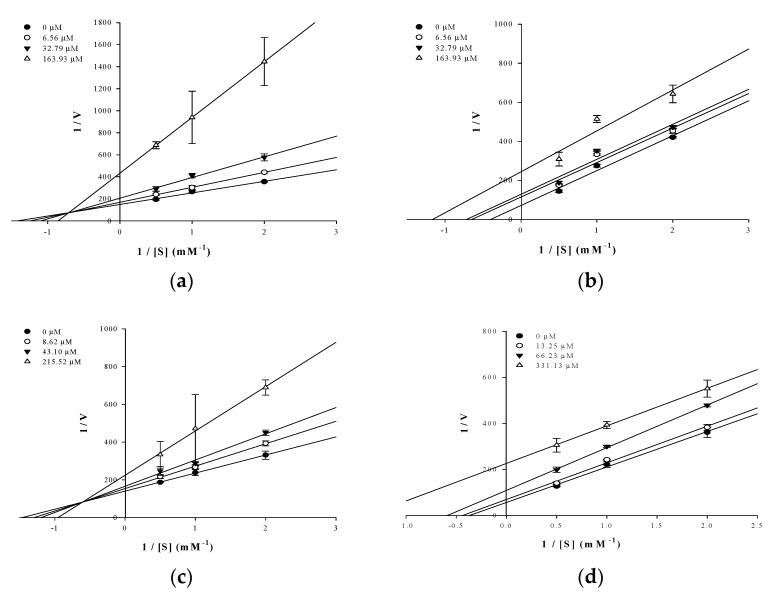
Lineweaver–Burk plots for the inhibition of PTP1B by the flavonoids. The results showed the effects of the presence of different concentrations of the flavonoids (0 μM (●), 6.56 μM (○), 32.79 μM (▼) and 163.93 μM (△) for hesperidin (**a**) and neohesperidin (**b**); 0 μM (●), 8.62 μM (○), 43.10 μM (▼) and 215.52 μM (△) hesperetin 5-*O*-glucoside (**c**) and 0 μM (●), 13.25 μM (○), 66.23 μM (▼) and 331.13 μM (△) hesperetin (**d**).

In an attempt to explain the mode of enzymatic inhibition of active flavonoids, kinetic analysis was performed at different concentrations of the substrate pNPP for PTP1B and the inhibitor. The type of inhibition and inhibition constants (*K*_i_) of flavonoids were investigated using Lineweaver–Burk and Dixon plots (Figure 2a–d and Figure 3a–d). Each line of inhibitors intersected at the xy-side, indicating that they are mixed-type inhibitors. Therefore, hesperidin and hesperetin 5-*O*-glucoside exhibited mixed-type inhibition with K*_i_* values of 50.02 and 81.62 μM, respectively, whereas neohesperidin and hesperetin displayed uncompetitive-type inhibition with K*_i_* values of 154.28 and 295.14 μM, respectively (Table 1).

### 2.3. Molecular-Docking Study of the Inhibition of PTP1B by Flavonoids

**Figure 4:**
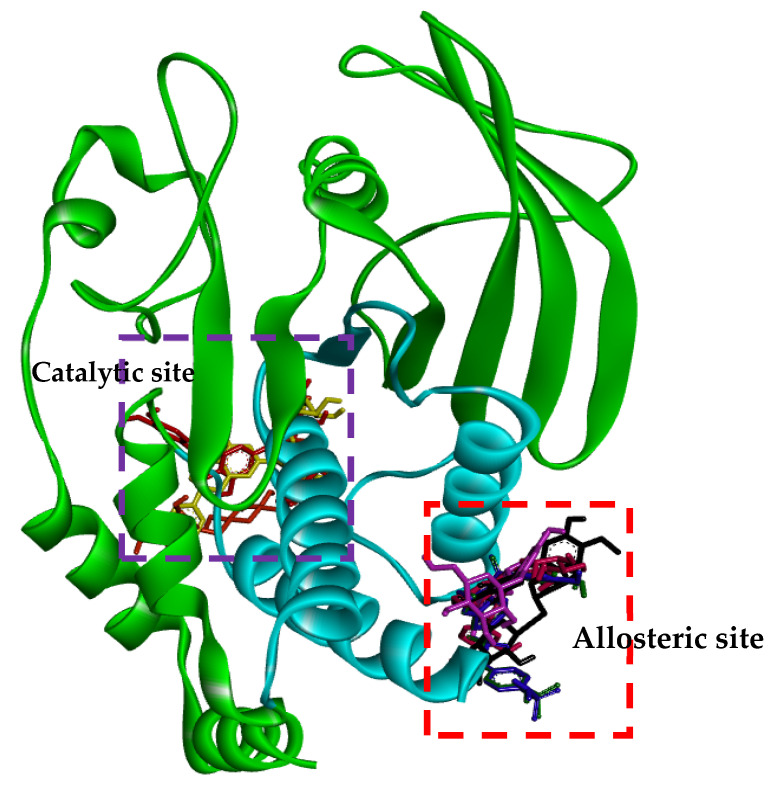
Molecular docking of PTP1B inhibition by compounds (compound **23**, compound **2**, hesperidin, neohesperidin, hesperetin 7-*O*-glucoside, hesperetin 5-*O*-glucoside and hesperetin). The tested compounds (compound **23**, compound **2**, hesperidin, neohesperidin, hesperetin 7-*O*-glucoside, hesperetin 5-*O*-glucoside and hesperetin) are represented by orange, blue, yellow, red, lavender, purple and green colored structures, respectively.

Molecular docking is one of the most frequently used approaches in structure-based drug design because of its ability to predict, with a substantial degree of accuracy, the conformation of small-molecule ligands within the appropriate target binding site. To further understand the mechanisms of action of the flavonoids with inhibitory activity against PTP1B, we performed molecular docking simulations. Specifically, we used AutoDock vina to predict and investigate the interactions between five flavonoids (hesperidin, neohesperidin, hesperetin 7-*O*-glucoside, hesperetin 5-*O*-glucoside and hesperetin) and PTP1B, whereas 3-({5-[(*N*-acetyl-3-{4-[(carboxycarbonyl)(2-carboxyphenyl)amino]-1-naphthyl}-L-alanyl)amino]pentyl}oxy)-2-naphthoic acid (compound **23**) and 3-(3,5-dibromo-4-hydroxy-benzoyl)-2-ethyl-benzofuran-6-sulfonic acid (4-sulfamoyl-phenyl)-amide (compound **2**), with structures defined in Section 4.4, were considered as the standard ligands for validating the AutoDock vina results (Figure 4).

**Figure 5:**
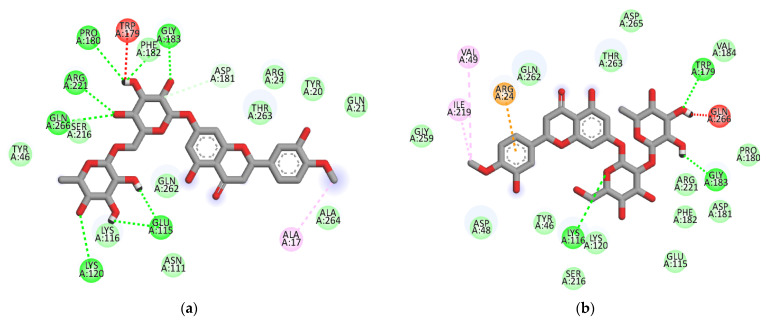

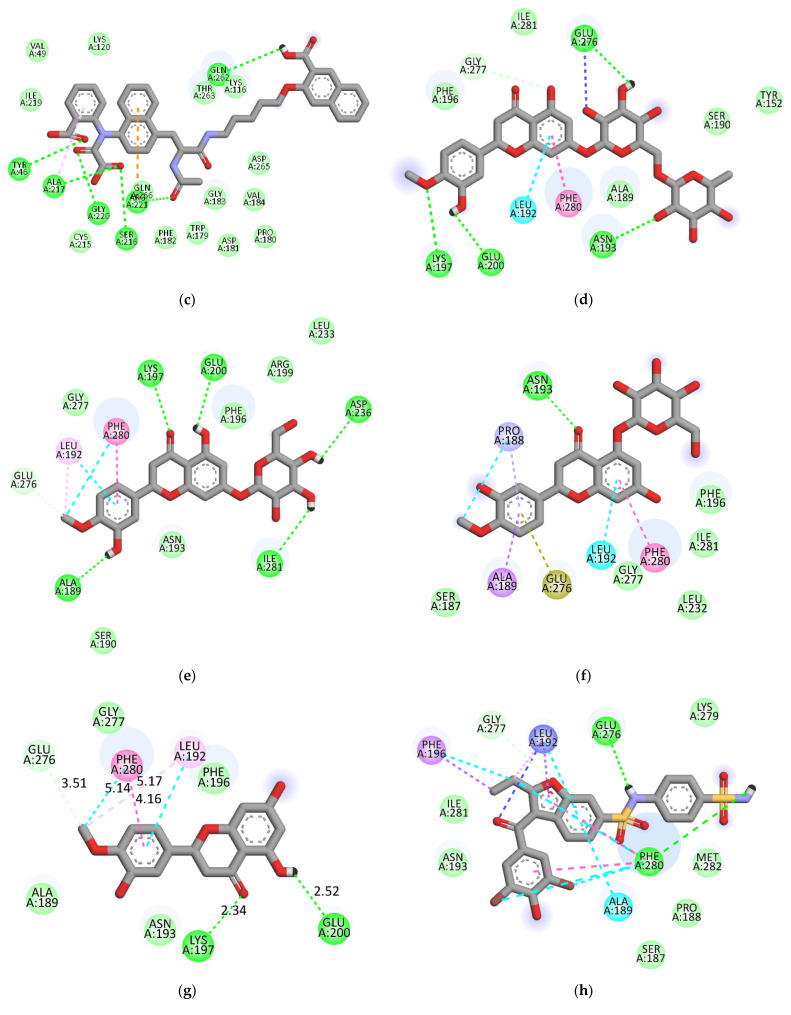

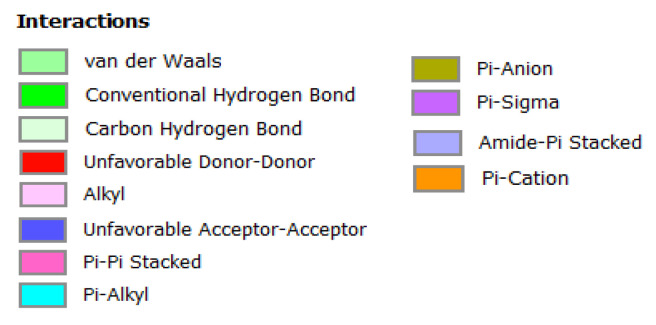
2D molecular docking models of the catalytic site hesperidin (**a**), neohesperidin (**b**) and compound **23** (**c**), and allosteric site hesperidin (**d**), hesperetin 7-*O*-glucoside (**e**), hesperetin 5-*O*-glucoside (**f**), hesperetin (**g**) and compound **2** (**h**) for PTP1B inhibition.

The ligand-enzyme complexes with flavonoids or compound **23** and compound **2** were stably posed in the catalytic and allosteric pockets of PTP1B by AutoDock vina. As illustrated in Figure 5a, hesperidin exhibited −8.4 kcal/mol binding affinity to the catalytic site of PTP1B. Hesperidin binds to PTP1B via the formation of eight hydrogen bonds, as shown in Figure 5a and Table 2. In particular, the hesperidin sugar moieties were observed to form hydrogen bond interactions with the Glu115, Lys120, Arg221, Phe182, Pro180, Gln266, and Gly183 residues of PTP1B. Moreover, the interacting methoxy group formed single alkyl bond interactions with Ala17. Some other interactions like unfavorable donor-donor with Trp179 and carbon-H bonds with Asp181 were observed. As shown in Figure 5b and Table 2, neohesperidin exhibited a binding affinity of −7.7 kcal/mol for PTP1B. Moreover, the neohesperidin sugar moieties formed three hydrogen bonds interactions with Lys116, Gly183, and Trp179. The neohesperidin sugar moiety was observed to form unfavorable donor-donor bond interaction with the Gln266 residue of PTP1B. Alkyl linkages were noticed with Val49 and Ile219 and π-cation linkage with Arg24. The molecular modeling of cognate ligand, compound **23** of protein tyrosine phosphatase 1B showed a network of π-cation and π-alkyl interactions and hydrogen bonding. As shown in Figure 5c, the amino acid residues six involve in hydrogen bonds were Tyr46, Ser216, Ala217, Gly220, Gln262, and Arg221. While π-cation with Gln266, and π-alkyl with Ala217. Moreover, hesperidin also bound to the allosteric site of PTP1B, showing considerable binding affinity (−7.9 kcal/mol) and illustrating two H-bonds with Glu276 and Asn193, with sugar moieties, whereas the hydroxyl and methoxy groups formed two hydrogen bond interactions with Glu200 and Lys197 (Figure 5d). In addition, π-alkyl interaction by Leu192, and unfavorable acceptor-acceptor interactions with Glu276, and π-π stacked with Phe280, and carbon-H bond with Gly277 were noticed. Furthermore, hesperetin 7-*O*-glucoside formed five hydrogen bonds with PTP1B and exhibited a binding affinity of −8.0 kcal/mol, as shown in Figure 5e and Table 2, respectively. The Asp236 and Ile281 residues were involved in two hydrogen bonds with the hesperetin 7-*O*-glucoside sugar moieties, whereas the hydroxyl and methoxy groups formed three hydrogen bond interactions with Ala189, Glu200, and Lys197. While carbon-H bond with Gln276 and π-π stacked with Phe280. Moreover, π-alkyl with Phe280 and Leu192 and alkyl linkages with Leu192 were observed (Figure 5e).

**Figure 6:**
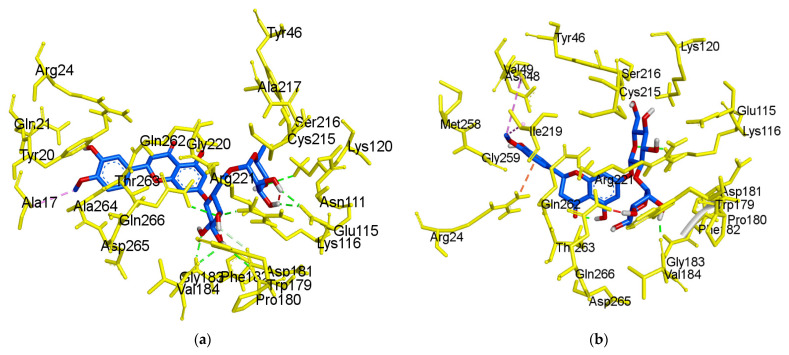

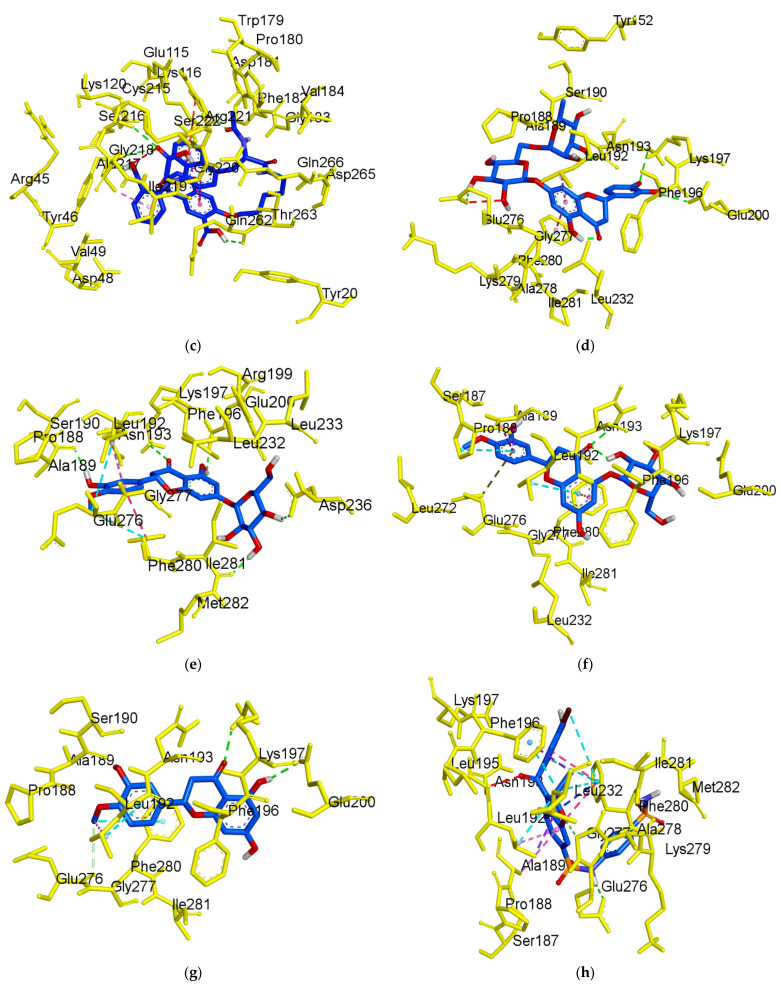
3D molecular docking models of the catalytic site hesperidin (**a**), neohesperidin (**b**) and compound **23** (**c**), and allosteric site hesperidin (**d**), hesperetin 7-*O*-glucoside (**e**), hesperetin 5-*O*-glucoside (**f**), hesperetin (**g**) and compound **2** (**h**) for PTP1B inhibition.

As illustrated in Figure 5f, hesperetin 5-*O*-glucoside exhibited a −8.3 kcal/mol binding affinity to PTP1B. The Asn193 residue was involved in a hydrogen bond with the hesperetin 5-*O*-glucoside ketone group, as shown in Table 2. Some amino acids showed π-alkyl interactions like Pro188 and Leu192. Other exhibited π-π stacked interactions with Phe280 and π-anion with Glu276. Additionally, π-sigma interaction was noticed with Ala189 and amide- π-stacked with Pro188. As shown in Figure 5g and Table 2, hesperetin exhibited a binding affinity of −7.6 kcal/mol for PTP1B. The hesperetin interactions of hydroxyl and ketone groups formed two hydrogen bond interactions with Glu200 and Lys197, respectively. Likewise, amino acids responsible for π-alkyl interactions were Phe280 and Leu192 and alkyl linkage with Leu192. In addition, hesperetin showed π-π stacked interaction with Phe280 and carbon-H with Glu276. On the other hand, the Phe280 and Glu276 enzyme residues participated in hydrogen-bonding interactions with compound **2** (Figure 5h). Some other interactions like unfavorable acceptor-acceptor with Leu192 and π-sigma with Phe196 and Leu192 were observed. Compound **2** made π-π stacked interactions with Phe280 and alkyl with Leu192 and π-alkyl linkage with Phe196, Leu192, Ala189 and Phe280. As shown in Figure 5h and Table 2, the binding energies of compound **2** were negative −8.8 kcal/mol, indicating that the additional hydrogen bonding might stabilize the open form of the enzyme and potentiate tighter binding to the PTP1B active site, resulting in more effective PTP1B inhibition. As shown in Figure 6a–h, the 3D interaction diagrams of flavonoids and we observed various van der Waals interactions between flavonoids and PTP1B residues that further stabilized the protein-ligand interaction.

## 3. Discussion

Various phytochemicals derived from nature, such as flavonoids, have attracted greater attention due to their numerous health advantages. The dietary flavonoids, especially their glycosides, are the most vital phytochemicals in diets and are of great general interest due to their diverse bioactivity [12,35]. The natural flavonoids almost all exist as their *O*-glycoside or *C*-glycoside forms and the bioavailability, metabolism, and biological activity of flavonoids depend upon the configuration, the total number of sugar moieties, and substitution of functional groups about their nuclear structure [12,35,36]. Recent studies have been demonstrated that the most reactive hydroxyl groups (7-OH or 5-OH in flavones) in flavonoids are generally glycosylated [35]. Glycosylation increases solubility in the aqueous cellular environment and protects the reactive hydroxyl groups from autooxidation [35,37]. It seems as though *O*-glycosylation can enhance certain types of biological benefits, including anti-HIV, anti-tyrosinase, anti-rotavirus, antistress, anti-obesity, anticholinesterase, antiadipogenic, and antiallergic activities [35]. Moreover, several epidemiological and human intervention studies suggest increased consumption of glycosylated flavonoid-rich foods is associated with a reduced risk of type 2 diabetes [12]. Generally, when glycosides are formed, the glycosidic linkage is normally located at position 7 or 5, and the carbohydrate can be L-rhamnose, D-glucose, glucose rhamnose, D-galactose, or L-arabinose [35,38].

In the present study, hesperetin (a flavanone) and its glycosylated derivatives, hesperidin, neohesperidin, hesperetin 7-*O*-glucoside, and hesperetin 5-*O*-glucoside, were characterized for their roles in diabetes, and the variation in activity corresponding to their structure was evaluated. PTP1B and α-glucosidase are two key inhibitory assays that were used to assess the anti-diabetic potentials of the hesperetin derivatives. Among hesperetin and its glycosylated derivatives, the most potent inhibitor of PTP1B was found to be hesperetin 5-*O*-glucoside, a single-glucose-containing flavanone glycosidase with an IC_50_ value of 37.14 ± 0.07 μM (Table 1). On the contrary, hesperetin 7-*O*-glucoside (7-position glucoside) drastically abolished inhibitory activity against PTP1B. In addition, aglycone hesperetin, which lacks a sugar moiety, showed weak inhibitory activity against PTP1B. Based on these observations, it was clear that the presence of a sugar moiety at the C-5 position is very important for PTP1B inhibitory activity. On the other hand, both hesperidin (hesperetin 7-*O*-rutinoside) and neohesperidin (hesperetin 7-*O*-neohesperidoside) contain two disaccharides but exhibited different levels of potency against PTP1B, due to the different positions of the glycosidic linkage. Hesperidin sugars consist of the α-1,6 interglycosidic linkage and showed significant inhibitory activity against PTP1B (IC_50_ = 58.15 ± 4.18 μM), whereas neohesperidin sugar moieties consist of α-1,2 interglycosidic bonds and display weak inhibitory activity against PTP1B (IC_50_ = 143.63 ± 3.04 μM). Thus, based on these results, it was clear that the position of the sugar moiety to be appears important for PTP1B inhibition. Interestingly, all flavonoids were inactive as observed in the α-glucosidase inhibitory assay, hinting that this pathway may be less important for the bioactivity of flavonoids. Thus, the main approach of the present study was to analyze the selective inhibition of PTP1B by naturally occurring flavonoids. Research on PTP1B inhibitors as potential therapeutic options for the treatment of DM and obesity has reached a peak, as approximately 300 PTP1B inhibitors have been developed from a variety of natural sources, with the largest representation from flavonoids [11,39]. Nevertheless, finding a potent and selective molecule, with good oral availability, is still a challenge to be overcome.

Naturally occurring flavonoids co-exist as aglycone and glycoside conjugates, and though certain aglycones have exhibited potent activity against PTP1B, their toxicity at certain concentrations has led researchers to shift their attention to flavonoid glycosides. It was previously suggested that the deglycosylation of flavonoid mono- or di-glucosides, such as prunin, quercetin 4-glucoside, naringin, narirutin, hesperidin, and naringenin greatly facilitated the expression of their bioactivities [12,35,40,41]. Other studies also reported that the influence of the glycosylation of flavonoids affects anti-diabetic activity, such as in the inhibition of advanced glycation end-product formation depending on the conjugation position and the class of sugar moiety [37,42,43,44]. Moreover, several in vitro and animal studies show that glycosylated flavonoids may have a role in type 2 diabetes therapy by modulating hepatic glucose homeostasis and insulin sensitivity [12]. Moreover, the glycosylation of flavonoids also affected the inhibition against aldose reductase depending on the number and types of sugar moiety and conjugation sites, as well as types of glycosylation [35,42,44,45,46,47,48]. Additionally, the in vivo bioactivity of flavonoids largely depends on their bioavailability, which can vary widely due to the structural diversification of these compounds. According to bioavailability studies, the majority of the flavanones undergo Phase II metabolism and the methylated, sulfated, or glucuronidated metabolites are the primary compounds found in the circulation [12].

The type of inhibition of the active hesperetin derivatives against PTP1B activity was studied using Lineweaver–Burk plots. Analysis of these plots at different substrate and fixed inhibitor concentrations indicated that hesperidin and hesperetin 5-*O*-glucoside showed a mixed-type and hesperetin and neohesperidin an uncompetitive-type inhibition mechanism. As observed in Figure 3a,c, hesperidin and hesperetin 5-*O*-glucoside presented mixed-type inhibition, since the K_m_ value increased while the V_max_ decreased. This means that both the inhibitor and substrate can be attached to the enzyme, with the inhibitor attached outside of the active site of PTP1B. In this inhibition type, the enzyme binding activity is affected by the binding of the substrate or inhibitor. This indicates that the inhibitor can bind to both the allosteric site of the free enzyme and the substrate–enzyme complex with similar affinity. After kinetic studies, the binding mode and the type of interactions between the flavonoids and the PTP1B were analyzed by molecular docking studies.

The crystal structure of the *human*-PTP1B (PDB ID: 1T49, 1NNY) complex allows the decoding of the active site residues (His214-Cys215-Ser216-Ala217-Gly218-Ile219-Gly220-Arg221) which bind to the phosphorylated tyrosine moiety of the substrate proteins [49]. In addition, a secondary binding site, comprised of amino acid residues (Arg24, Arg254, Gln262 and a few others, such as Tyr46, Asp48, Val49, Met258), has also been reported adjacent to the main active site [50]. The hydrophobic pocket site of PTP1B consists of Tyr46, Asp48, Lys120, Gln262, and Gln266 residues [49]. The molecular docking models of flavonoids, along with compound **23** and compound **2** as the standard ligands, are illustrated in Figure 4 and Table 2. The ligand–enzyme complexes with flavonoids/or compound **23** and compound **2** were stably posed in the same pocket of PTP1B by Autodock vina. The binding energies of flavonoids were −8.4, −7.9, −7.7, −8.0, −8.3, and −7.6 Kcal/mol for hesperidin (catalytic site), hesperdin (allostieric site), neohesperidin, hesperetin 7-*O*-glucoside, hesperetin 5-*O*-glucoside, and hesperetin respectively. The PTP1B-hesperidin inhibitor complex had the highest binding energy and the hydroxyl groups of hesperidin showed H-bond interactions with the important residues Arg221, Gln266, and Pro180 of the catalytic and hydrophobic sites of PTP1B, respectively. The sugar moiety −OH groups of neohesperidin formed H-bonds with Gly183, Trp179 and Lys116 at the catalytic active site. Hesperetin 7-*O*-glucoside showed negative binding energies and high proximity to PTP1B residues, Ile281, Ala189, Asp236, Lys197, and Glu200 in the pocket site. Hesperetin formed two H-bond with two important residues of Lys197 and Glu200. This study also demonstrates that, at the allosteric site of PTP1B, there is a prominent interaction of hesperetin 5-*O*-glucoside with the key residue Asn193 and confirms the mixed-type allosteric inhibition of PTP1B. The phenyl ring of hesperetin 5-*O*-glucoside is also stabilized by the π–π interactions, along with the Phe280 and π-sigma with Ala189 residue.

We previously found that prunin is a competitive inhibitor that binds directly to the catalytic active site of PTP1B in the presence of a glucose moiety [51]. Recently, we also reported that naringenin derivatives were tested as PTP1B inhibitors, and that hydrophobic and hydrogen bonding interactions are important for the strength of the protein-ligand interaction, as is the positioning of the inhibitors in the catalytic pocket [41]. Even though hesperidin has a higher number of H-bonds and binding energies that are greater than that of hesperetin 5-*O*-glucoside, which has fewer H-bonds. However, hesperetin 5-*O*-glucoside binds to the key residues (Leu192, Asn193, Phe280, Pro188, Ala189, and Glu276) and is the best fit to the PTP1B allosteric site cavity.

Hesperetin 5-*O*-glucoside, a flavanone glycoside isolated from *Prunus davidiana* that is reported to have biological activities, including antihyperlipidemic and hypocholesterolemic effects [30], and antioxidant activity by inhibition of hydroxyl radicals (OH), reactive oxygen species (ROS), and the scavenging of peroxynitrites [31]. Nevertheless, this is the first work demonstrating the anti-diabetic activity of hesperetin 5-*O*-glucoside via the inhibition of PTP1B and α-glucosidase. Here we found distinct differences in the IC_50_ values of hesperetin derivatives against PTP1B and identified hesperetin 5-*O*-glucoside as the best inhibitor. This finding depicts hesperetin 5-*O*-glucoside as more effective than other hesperetin derivatives, which is our new finding. The novel inhibitory activity of hesperetin 5-*O*-glucoside, which contains a single glucose molecule at the C-5 position, likely has a favorable conformation and allows it to fit suitably within the allosteric site of the enzyme. These results suggest that hesperetin 5-*O*-glucoside possesses potential anti-diabetic activity by the inhibition of PTP1B and holds great promise for the treatment of DM.

## 4. Materials and Methods

### 4.1. Chemicals and Reagents

Hesperidin, neohesperidin, hesperetin, hesperetin 7-*O*-glucoside, *p*-nitrophenyl phosphate (pNPP), and ethylenediaminetetraacetic acid (EDTA) were purchased from Sigma-Aldrich. PTP1B (human recombinant) was purchased from Biomol International LP (Plymouth Meeting, PA, USA), and dithiothreitol (DTT) was purchased from Bio-Rad Laboratories (Hercules, CA, USA). All other chemicals and solvents used were purchased from E. Merck, Fluka, and Sigma-Aldrich, unless otherwise stated.

### 4.2. PTP1B Inhibitory Assay

The PTP1B inhibitory activity was evaluated using pNPP according to the work of Jung et al. [41]. In each well of a 96-well plate (each with a final volume of 100 µL), 40 µL of PTP1B enzyme [0.5 units diluted with a PTP1B reaction buffer containing 50 mM citrate (pH 6.0), 0.1 M NaCl, 1 mM EDTA, and 1 mM DTT] was added with or without the sample dissolved in 10% DMSO. The plate was preincubated at 37 °C for 10 min and then 50 µL of 2 mM pNPP in the PTP1B reaction buffer was added. Following incubation at 37 °C for 20 min, the reaction was terminated by the addition of 10 M NaOH. The amount of *p*-nitrophenyl produced after enzymatic dephosphorylation of pNPP was estimated by measuring the absorbance at 405 nm using a microplate spectrophotometer (Molecular Devices). The nonenzymatic hydrolysis of 2 mM of pNPP was corrected by the measured increase in absorbance at 405 nm obtained in the absence of the PTP1B enzyme. The inhibition percentage was obtained using the following equation: % inhibition = (Ac − As)/Ac × 100, where Ac is the absorbance of the control and As is the absorbance of the sample. Ursolic acid was used as a positive control.

### 4.3. Kinetic Parameters of Hesperetin Derivatives in PTP1B Inhibition

To determine the kinetic mechanism, two kinetic methods using Lineweaver–Burk and Dixon plots were complementarily used. Using Lineweaver–Burk and Dixon plots, the PTP1B inhibition mode was determined at various concentrations of *p*-NPP substrate (0.5, 1.0 and 2.0 mM) in the absence or presence of different test flavonoid concentrations (0, 6.56, 32.79 and 163.93 µM for hesperidin and neohesperidin; 0, 8.62, 43.10 and 215.52 µM for hesperetin 5-*O*-glucoside; 0, 13.25, 66.23, 331.13 µM for hesperetin). The enzymatic inhibitions of the test flavonoids were evaluated by monitoring the effects of different concentrations of the substrates in the Dixon plots (single reciprocal plot). The enzymatic procedure consisted of the same, aforementioned, PTP1B assay method. The inhibition constants (*K_i_*) were determined by interpretation of the Dixon plots, where the value of the *x*-axis implies -*K_i_*.

### 4.4. Molecular Docking Simulation in PTP1B Inhibition

The X-ray crystallographic structure of PTP1B, with its selective inhibitor 3-(3,5-dibromo-4-hydroxy-benzoyl)-2-ethyl-benzofuran-6-sulfonic acid (4-sulfamoyl-phenyl)-amide (compound **2**) (PDB ID: 1T49) and the 3D structure of catalytic inhibitor 3-({5-[(*N*-acetyl-3-{4-[(carboxycarbonyl)(2-carboxyphenyl)amino]-1-naphthyl}-L-alanyl)amino]pentyl}oxy)-2-naphthoic acid (compound **23**) were obtained from the RCSB Protein Data Bank (PDB ID: 1NNY) [52,53] website at resolutions of 1.9 Å and the PubChem Compound (NCBI) with compound CID of 447410, respectively. The binding PTP1B inhibitor and water molecules were removed from the structure for the docking simulation using Accelrys Discovery Studio 4.1 (Accelrys, Inc., San Diego, CA, USA). The 3D structures of hesperidin, neohesperidin, hesperetin 7-*O*-glucoside, hesperetin 5-*O*-glucoside, and hesperetin were obtained from the PubChem Compound (NCBI), with compound CIDs of 10621, 442439, 147394, 18625123, and 72281, respectively, and were protonated (pH 7.0) using MarvinSketch program (ChemAxon, Budapest, Hungary). The automated docking simulation was performed using AutoDock Tool (ADT) to assess the appropriate binding orientations and conformations of PTP1B with the different compounds. A Lamarckian genetic algorithm method implemented in AutoDock vina was employed. For docking calculations, Gasteiger charges were added by default, the rotatable bonds were set by the ADTs, and all torsions were allowed to rotate. The grid maps were generated by the Autogrid program where the grid box size of 80 × 80 × 80 had a default spacing of 0.375 Å. The respective X, Y, Z coordinates of the center were 56.019, 31.367 and 22.486, respectively. The docking protocol for a rigid and flexible ligand docking consisted of 10 independent genetic algorithms, while the other parameters used were default parameters of the ADT. The binding aspects of the PTP1B residues and their corresponding binding affinity scores were regarded as the best molecular interaction. The results were analyzed using UCSF Chimera [54], while the hydrogen bonds and van der Walls interaction residues were visualized by the Discovery Studio 2016 Client.

### 4.5. Statistics

All results are expressed as the mean ± SEM of triplicate samples. The statistical significances were analyzed using the one-way ANOVA and Student’s *t*-test (Systat Inc., Evanston, IL, USA), and were noted at *p* < 0.05.

## 5. Conclusions

In this work, a promising flavonoid scaffold was found as a potential effective PTP1B inhibitor for the treatment of DM. Hesperetin 5-*O*-glucoside is a potent PTP1B inhibitor with a mixed inhibitory mechanism. Hesperetin 5-*O*-glucoside, with its single sugar moiety, displayed reasonable binding energy and the highest binding affinity for PTP1B inhibition. The structure-activity relationship studies indicated that the single sugar moiety at the C-5 position was crucial for the activity against PTP1B inhibition. Our results indicated that flavanones containing one sugar moiety at the 5-position, such as hesperetin 5-*O*-glucoside, best fit the PTP1B allosteric site cavity for PTP1B inhibition. Further in vivo and cellular-based studies are needed to help clarify the detailed mechanism of action of these flavonoids in the brain membrane and other organs.

## Figures and Tables

**Table 1:** Inhibitory activities of hesperetin derivatives on the tyrosine phosphatase 1B (PTP1B) protein.

Compound	PTP1B		
	IC_50_ (μM) ^a^	Type of Inhibition^b^	K*_i_* (μM) ^c^
Hesperidin	58.15 ± 4.18	Mixed	50.02
Neohesperidin	143.63 ± 3.04	Uncompetitive	154.28
Hesperetin 7-*O*-glucoside	>300	-	-
Hesperetin 5-*O*-glucoside	37.14 ± 0.07	Mixed	81.62
Hesperetin	288.01 ± 7.98	Uncompetitive	295.14
Ursolic acid ^d^	9.65 ± 0.01		

^a^ The 50% inhibitory concentration (IC_50_) values (μM) were calculated from a log dose inhibition curve and as the mean ± S.E.M of triplicate experiments. ^b^ Determined by Lineweaver–Burk plots. ^c^ Determined by Dixon plots. ^d^ Used as positive control.

**Table 2:** Binding site residues and docking scores of flavonoids in the tyrosine phosphatase 1B protein using the AutoDock vina program.

Compound	Binding Energy ^a^ (kcal/mol)	H-Bonding Interacting Residues	Other Interactions
Hesperidin ^b^	−8.4	Lys120 (2.87 Å), Glu115 (2.04 and 2.12 Å), Arg221 (2.00 Å), Gln266 (2.53 Å), Pro180 (2.79 Å), Gly183 (2.06 and 2.53 Å)	Asp181 (C-H 3.61 Å), Trp179 (Unfavorable donor-donor 2.15 Å) and Ala17 (Alkyl 4.03 Å)
	−7.9	Lys197 (3.01 Å), Glu200 (2.39 Å), Asn193 (2.10 Å), Glu276 (2.70 Å)	Leu192 (π-Alkyl 4.41 Å), Phe280 (π-π stacked 3.81 Å), Glu276 (Unfavorable Aceptor-Aceptor 2.95 Å), Gly277 (C-H 3.10 Å)
Neohesperidin	−7.7	Lys116 (2.59 Å), Trp179 (1.87 Å), Gly183 (1.88 Å)	Ile219 (Alkyl 5.09 Å), Val49 (Alkyl 5.11 Å), Gln266 (Unfavorable donor-donor 1.13 Å), Arg24 (π-cation 3.67 Å)
Hesperetin 7-*O*-glucoside	−8.0	Ala189 (2.07 Å), Ile281 (2.16 Å), Asp236 (2.78 Å), Glu200 (2.87 Å), Lys197 (2.84 Å)	Glu276 (C-H 3.67 Å), Leu192 (Alkyl 5.05 Å), Leu192 (π-Alkyl 4.53 Å), Phe280 (π-Alkyl 5.18 Å), Phe280 (π-π stacked 4.12 Å)
Hesperetin 5-*O*-glucoside	−8.3	Asn193 (2.46 Å)	Phe280 (π-π stacked 4.00 Å), Leu192 (π-Alkyl 4.5 Å), Pro188 (π-Alkyl 4.59 Å), Ala189 (π-sigma 3.51 Å), Pro188 (amide π-stacked 4.77 Å), Glu276 (π-anion 4.26 Å)
Hesperetin	−7.6	Lys197 (2.34 Å), Glu200 (2.52 Å)	Leu192 (π-Alkyl 4.53 Å), Phe280 (π-Alkyl 5.14 Å), Phe280 (π-π stacked 4.16 Å), Leu192 (Alkyl 5.17 Å), Glu276 (C-H 3.51 Å)
Compound 2 ^c^ (Allosteric inhibitor)	−8.8	Glu276 (2.27 Å), Phe280 (2.57 Å)	Phe280 (π-Alkyl 5.48, 5.41 and 5.02 Å), Ala189 (π-Alkyl 4.90 Å), Leu192 (π-Alkyl 4.85 Å), Phe196 (π-Alkyl 3.93 Å), Leu192 (Unfavorable Aceptor-Aceptor 2.69 Å), Leu192 (π-sigma 3.82 Å), Phe196 (π-sigma 3.74 Å), Leu192 (Alkyl 4.40 Å), Phe280 (π-π stacked 4.11 and 4.08 Å), Gly277 (C-H 3.38 Å)
Compound 23 ^c^ (Catalytic inhibitor)	−8.6	Tyr46 (2.55 Å), Gly220 (2.32 Å), Ala217 (2.72 Å), Ser216 (2.65 Å), Arg221 (2.25 Å), Gln262 (2.41 Å)	Gln266 (π-cation 4.65 Å), Ala217 (Alkyl 3.99 Å)

^a^ Estimated the binding free energy of the ligand-receptor complex. ^b^ Hesperidin showed both types of catalytic (upper) and allosteric (lower) inhibition. ^c^ Compound **23** (3-({5-[(*N*-acetyl-3-{4-[(carboxycarbonyl)(2-carboxyphenyl)amino]-1-naphthyl}-L-alanyl)amino]pentyl}oxy)-2-naphthoic acid) and compound **2** (3-(3,5-dibromo-4-hydroxy-benzoyl)-2-ethyl-benzofuran-6-sulfonic acid (4-sulfamoyl-phenyl)-amide) were used as positive ligands.

## Data Availability

This manuscript does not have any data-sharing criteria.

## Sample Availability

Samples of the compounds are available from the authors on request.
